# Vitamin D status as a predictor for liver transplant outcomes

**DOI:** 10.1038/s41598-023-48496-5

**Published:** 2023-11-29

**Authors:** Danial Fotros, Mohammadhassan Sohouli, Zahra Yari, Hanie Sakhdari, Mojtaba Shafiekhani, Hamed Nikoupour, Mohammad Amin Jafarzadeh, Keynoosh Jafari, Seyedeh Sara Afiatjoo, Seyed Amirreza Fatemi, Mahmoud Amiri, Hesameddin Eghlimi, Amirhassan Rabbani, Nasrin Broumandnia, Ghazaleh Mahdavi Mazdeh, Ali Jafarian, Azita Hekmatdoost

**Affiliations:** 1 Department of Clinical Nutrition and Dietetics, Faculty of Nutrition and Food Technology, National Nutrition and Food Technology Research Institute, Shahid Beheshti University of Medical Sciences, Tehran, Iran; 2 Department of Nutrition Research, National Nutrition and Food Technology Research Institute, Shahid Beheshti University of Medical Sciences, Tehran, Iran; 3 Student Research Committee, Babol University of Medical Science, Babol, Mazandaran Iran; 4 Shiraz Organ Transplant Center, Abu-Ali Sina Hospital, Shiraz University of Medical Sciences, Shiraz, Iran; 5 Shiraz Transplant Research Center, Shiraz University of Medical Sciences, Shiraz, Iran; 6 Student Research Committee, Shiraz University of Medical Sciences, Shiraz, Iran; 7 Department of Medical-Surgical Nursing, School of Nursing and Midwifery, University of Medical Sciences, Tehran, Iran; 8 Department of General Surgery, Ayatollah Taleghani Hospital, Shahid Beheshti University of Medical Science, Tehran, Iran; 9 Urology and Nephrology Research Center, Shahid Beheshti University of Medical Sciences, Tehran, Iran; 10 Division of Hepatopancreatobiliary and Liver Transplantation Surgery, Imam Khomeini Hospital Complex, Tehran, Iran

**Keywords:** Biochemistry, Biomarkers, Gastroenterology, Risk factors

## Abstract

It is well known that vitamin D plays a pivotal role in immune system modulation; however, its role in liver transplantation (LT) has not yet been well elucidated. This study aimed to assess the association between vitamin D status and LT outcomes. This retrospective cohort study was conducted on 335 registered cirrhotic patients with end-stage liver disease (ESLD) who underwent LT during 2019–2021 and had measurement of serum vitamin D before LT. The association of vitamin D levels before LT with the odds of acute cellular rejection (ACR) and risk mortality was assessed by applying logistic and cox regression, respectively. The mean MELD-Na and serum level of vitamin D were 20.39 ± 9.36 and 21.52 ± 15.28 ng/ml, respectively. In the final adjusted model, there was a significant association between vitamin D deficiency in the pre-transplant period and odds of ACR (odds ratio [OR] 2.69; 95% confidence interval [CI] 1.50–4.68). Although in the crude model, vitamin D deficiency in the pre-transplant period was significantly associated with an increased risk of mortality after two years of follow-up (Hazard ratio (HR) = 2.64, 95% CI 1.42–4.33), after adjustment for potential confounders, the association of vitamin D status and mortality became non-significant (HR = 1.46, 95% CI 0.71–3.00). The present study provides evidence that pre-transplant serum vitamin D levels may be a predictor for ACR in patients with cirrhosis undergoing LT.

## Introduction

Cirrhosis is the terminal stage of chronic liver disease, characterized by chronic inflammation and progressive fibrosis. This condition is primarily caused by chronic alcoholism, metabolic-associated fatty liver disease, and viral hepatitis^1^. In the United States, cirrhosis is the ninth leading cause of death, accounting for 1.2% of all deaths^2^. Globally, the incidence of liver cirrhosis increased by 16.7% from 2009 to 2019, rising from 1.8 million to 2.1 million cases^3^. End-stage liver disease (ESLD) is a culmination of chronic liver disease progressing to decompensated cirrhosis and hepatocellular failure^1^. These patients may benefit from liver transplantation (LT), which can provide a cure and improved long-term survival outlook^4^. However, it is often accompanied by acute cellular rejection (ACR), which occurs in 30–70% of cases^5–8^. LT outcomes may be affected by a number of factors, such as liver enzymes, cytomegalovirus (CMV) infections, biochemical parameters such as serum vitamin D levels, and dietary factors^9–12^.

Vitamin D plays a crucial role in bone metabolism^13^, gene expression in various tissues^14^, and calcium absorption in the gut^15^. Aside from that, there is some evidence that vitamin D may benefit individuals suffering from liver diseases due to its anti-inflammatory and immunomodulatory properties^16–19^. Interestingly, the prevalence of vitamin D insufficiency is extremely high among patients with ESLD who are eligible for transplantation, with up to 93% of these patients suffering from some degree of insufficiency^20,21^.

Furthermore, several studies have shown that vitamin D can reduce the risk of ACR in solid organ transplants such as kidneys and lungs^22,23^. Consequently, vitamin D may contribute to improved outcomes in LT from a nutritional perspective^11^.

To the best of our knowledge, few studies have investigated the association between pre-transplant vitamin D and ACR or mortality in liver transplant recipients. In the present study, we aimed to examine the relationship between vitamin D status and ACR and mortality in liver transplant recipients. By examining this relationship, we hope to develop a better understanding of the role of vitamin D and its serum level correction in the successful management of LT.

## Materials and methods

### Study population

This retrospective cohort study examined the registration information of individuals with cirrhosis who underwent LT between 2019 and 2021 at Taleghani Hospital (Tehran) and Abu-Ali Sina Hospital (Shiraz). Patients with ESLD with complete lab results for vitamin D and other routine laboratory testing were eligible to enter the study. On the other hand, certain groups of patients were excluded from the study. These included individuals with acute liver failure, those who had undergone multiple organ transplants, and people who had experienced primary graft non-function (PNF). By setting these criteria, the study aimed to ensure that the results were obtained from a homogeneous group of individuals with similar characteristics to enhance the validity of the findings. Finally, three hundred thirty-five cases were considered for the follow-up in this study (Table 1). This study was conducted in accordance with the Declaration of Helsinki, and the ethics committee of National Nutrition and Food Technology Research Institute approved the protocol.

**Table 1:** Characteristics of all population study prior to liver transplantation.

Characteristics	
Age, y	40.22 ± 19.14
Male, n (%)	208 (62.1)
BMI ^b^, kg/m^2^	23.36 ± 4.84
MELD-Na	20.39 ± 9.36
Waiting time for transplantation, months	4.38 ± 6
Cause of cirrhosis, n (%)	
Viral	46 (13.7)
NASH	48 (14.3)
PSC/PBC	88 (26.3)
AIH	46 (13.7)
Genetic Causes	35 (10.4)
Other	71 (21.2)
Alcohol intake (yes), n (%)	19 (5.7)
Smoking (yes), n (%)	2 (0.6)
CMV positive (yes), n (%)	64 (19.1)
Hypertension (yes), n (%)	25 (7.5)
Diabetes millitus (yes), n (%)	76 (22.7)
Medical condition at the time of LT, n (%)	
ICU	10 (3)
Hospitalized	62 (18.5)
Home	263 (78.5)
ALT (U/L)	92.41 ± 311.02
AST (U/L)	120.51 ± 308.74
ALP (U/L)	497.09 ± 400.30
GGT (U/L)	142.38 ± 182.71
Albumin (g-dL)	3.38 ± 0.74
FBS (mg/dL)	113.52 ± 61.65
TC (mg/dL)	141.42 ± 88.22
TG (mg/dL)	120.45 ± 92.28
LDL-C (mg/dL)	84.01 ± 64.45
HDL-C (mg/dL)	33.04 ± 14.83
WBC (per microliter)	6695.41 ± 3842.47
PMN (%)	63.36 ± 15.34
Lymphocytes (%)	25.25 ± 13.76

BMI: body mass index; MELD-Na: Model For End-Stage Liver Disease; NASH: nonalcoholic steatohepatitis; PSC: primary sclerosing cholangitis; PBC: primary biliary cholangitis; AIH: autoimmune hepatitis; CMV: Cytomegalovirus; ICU: intensive care unit; ALT: alanine transaminase; AST: aspartate aminotransferase; ALP: alkaline phosphatase; GGT: gamma-glutamyl transferase; FBS: fasting blood sugar; TC: total cholesterol; TG: triglycerides; LDL-C: low-density lipoprotein cholesterol; HDL-C: high-density lipoprotein cholesterol; WBC: White Blood Cell; PMN: polymorphonuclear neutrophil.

### Data collection

First, demographic and clinical pathophysiology data of the patients were obtained, including age, gender, body mass index (BMI), cause of cirrhosis, medical conditions at the time of LT, history of diabetes/hypertension, smoking, Alcohol consumption, and the waiting time it took for the liver transplant to occur.

Additionally, other variables, including lab results, such as alanine transaminase (ALT), aspartate aminotransferase (AST), alkaline phosphatase (ALP), gamma-glutamyl transferase (GGT), fasting blood sugar, total cholesterol, triglycerides, low-density lipoprotein cholesterol (LDL-C), high-density lipoprotein cholesterol (HDL-C), white blood cells (WBC), and polymorphonuclear neutrophils (PMN) were collected based on the last laboratory result prior to LT. The final step was to collect data regarding ACR, mortality, and CMV infection after LT. Since this study was conducted at two centers, it is worth noting that the measurement methods were consistent in both centers. Moreover, after the transplant, the patients usually received a combination of antirejection medications, which include tacrolimus (0.02–0.03 mg/kg/day), mycophenolate mofetil (1–2 g/day, up to 3 g/day), and prednisolone (up to 20 mg/day).

### Vitamin D measurement

25-hydroxyvitamin D levels (25(OH)D) were measured along with other laboratory tests before LT. In this study, serum levels of vitamin D under 20 ng/ml were considered deficient, and ≥ 20 ng/ml as sufficient. For vitamin D, we used the most recent lab results available before LT, from which we also obtained other lab results.

### MELD and MELD-Na calculation

The Model for End-Stage Liver Disease (MELD) scores were calculated using the following formulas based on the last lab results before LT for bilirubin, creatinine, INR, and Na^24^. Serum sodium value was corrected in the range of 125–137 mEq/l, according to criteria determined by UNOS (United Network for Organ Sharing).

$$ \begin{aligned} MELD = & 3.78 \times \ln \left[ {serum\;bilirubin\left( {mg/dL} \right)} \right] + 11.2 \times \ln \left[ {INR} \right] \\ \quad & + 9.57 \times \ln \left[ {serum\;creatinine\left( {mg/dL} \right)} \right] + 6.43 \\ \end{aligned} $$

$$ MELD{ - }Na = MELD + 1.32x\left( {137 - Na} \right) - \left[ {0.033xMELD*\left( {137 - Na} \right)} \right] $$

### Outcomes identification

In clinical practice, ACR after LT is associated with allograft dysfunction, which is concurrently diagnosed by liver biopsy and histological analysis^25^. Accordingly, when there was suspicion that there may be an ACR reaction, a biopsy was performed in this study. Mortality was also monitored for two years following LT or until death occurred. In addition, CMV infection was detected and diagnosed by CMV antigen-positive peripheral leukocytes.

### Statistical analysis

After providing the normality of the distribution of the studied variables by Kolmogorov–Smirnov test, independent sample T-Test used to compare quantitative variables between the two groups, as well as Chi-square or fisher exact statistical test was also used for qualitative variables. The baseline characteristics were reported as mean ± standard deviation (SD) for quantitative variables, and number and percentages for qualitative variables. Regression model was used to examine the correlation between variables. The association of vitamin D levels before LT with the odds of ACR and risk of mortality was assessed by applying logistic and cox regression, respectively. The analyses were adjusted for probable confounders, e.g., age, sex, BMI, waiting time for transplantation, medical condition at the time of LT, causes of cirrhosis, smoking, alcohol, hypertension, diabetes mellitus, infection of CMV, MELD-Na, AST, ALT, PMN, WBC, and lymphocytes. All analyses were performed by SPSS 25.0 statistical software, and *P*-value less than 0.05 was considered statistically significant.

### Ethical approval

This study was approved by the research ethics committee of Shahid Beheshti University of Medical Sciences, Tehran, Iran. We confirm that all methods were performed in accordance with the relevant guidelines and regulations. Also, informed consent was obtained and signed from all patients.

## Results

The mean ± SD age of the study population was 40.22 ± 19.14 years. The mean ± SD BMI was 23.36 ± 4.84 kg/m^2^. About 62% of the participants were male, and the rest were female. The mean ± SD MELD-Na and serum vitamin D levels were 20.39 ± 9.36 and 21.52 ± 15.28 ng/ml, respectively. The cause of cirrhosis was mainly primary sclerosing cholangitis (PSC) and primary biliary cholangitis (PBC) (n = 88, 26.3%), nonalcoholic steatohepatitis (NASH) (n = 48, 14.3%), and viral or autoimmune hepatitis (AIH) (both causes: n = 46, 13.7%). The mean waiting time was 4.38 months. In addition, about 19% of the samples were infected with CMV after transplantation, and the prevalence of diabetes and hypertension in the study population was 22.7% and 7.5%, respectively (Table 1).

The baseline characteristics of the patients between two groups of subjects with sufficient and deficient levels of vitamin D in the pre-transplant stage are shown in Table 2. Compared to those with sufficient vitamin D levels, those with vitamin D deficiency had higher MELD-Na scores and waiting time for LT but lower albumin levels. In addition, the number of patients with CMV infection after LT was significantly higher in those with vitamin D deficiency. However, no significant differences were found for other characteristics between subjects with sufficient and deficient levels of vitamin D.

**Table 2:** Characteristics of vitamin-D-deficient and vitamin D-sufficient patients prior to liver transplantation.

	Groups, Mean ± SD		*P* value^a^
	Sufficiency (≥ 20 ng/ml)	Deficiency (< 20 ng/ml)	
Age, y	41.35 ± 18.98)	39.47 ± 19.25)	0.38
Male, n (%)	75 (56)	133 (66.5)	0.052
BMI ^b^, kg/m^2^	23.55 ± 4.93	23.23 ± 4.79	0.56
MELD-Na	18.39 ± 8.64	21.74 ± 9.60	**0.001**
Waiting time for transplantation, months	3.53 ± 3.04	4.96 ± 7.32	**0.035**
Causes of cirrhosis, n (%)			
Viral	10 (7.5)	36 (17.9)	0.089
NASH	17 (12.8)	31 (15.4)	
PSC/PBC	39 (29.3)	49 (24.4)	
AIH	23 (17.3)	23 (11.4)	
Genetic Causes	15 (11.3)	20 (10)	
Other	29 (21.8)	42 (20.9)	
Alcohol intake (yes), n (%)	8 (6)	11 (5.5)	0.847
Smoking (yes), n (%)	1 (0.7)	1 (0.5)	0.772
CMV positive (yes), n (%)	17 (12.8)	47 (23.5)	**0.015**
Hypertension (yes), n (%)	11 (8.3)	14 (7.1)	0.681
Diabetes millitus (yes), n (%)	39 (29.8)	37 (18.9)	**0.022**
Medical condition at the time of LT, n (%)			
ICU	1 (0.7)	9 (4.5)	**0.015**
Hospitalized	18 (13.4)	44 (21.9)	
Home	115 (85.8)	148 (73.6)	
ALT (mg/dl)	65.85 ± 63.27	110.07 ± 397.42	0.204
AST (mg/dl)	86.49 ± 65.05	143.08 ± 393.42	0.103
ALP (U/L)	485.86 ± 371.27	504.59 ± 419.34	0.677
GGT (U/L)	114 ± 195.91	140.28 ± 166.21	0.921
Albumin (g/dL)	3.48 ± 0.72	3.30 ± 0.75	**0.040**
FBS (mg/dl)	108.29 ± 47.10	117.73 ± 71.16	0.268
TC (mg/dl)	148.77 ± 97.76	132.87 ± 75.62	0.329
TG (mg/dl)	114.42 ± 78.2	127.69 ± 107.07	0.433
LDL-C (mg/dl)	88.55 ± 73.88	78.63 ± 51.26	0.407
HDL-C (mg/dl)	34.56 ± 13.83	31.27 ± 15.87	0.229
WBC (per microliter)	6520.92 ± 3685.36	6810.86 ± 3947.95	0.504
PMN (%)	61.99 ± 13.61	64.27 ± 16.36	0.199
Lymphocytes (%)	27.39 ± 12.69	23.83 ± 14.28	**0.025**

^a^Obtained from Independent sample T-Test for continuous variables and Chi-square for Categorical variables.

BMI: body mass index; MELD-Na: Model For End-Stage Liver Disease; NASH: nonalcoholic steatohepatitis; PSC: primary sclerosing cholangitis; PBC: primary biliary cholangitis; AIH: autoimmune hepatitis; CMV: Cytomegalovirus; ICU: intensive care unit; ALT: alanine transaminase; AST: aspartate aminotransferase; ALP: alkaline phosphatase; GGT: gamma-glutamyl transferase; FBS: fasting blood sugar; TC: total cholesterol; TG: triglycerides; LDL-C: low-density lipoprotein cholesterol; HDL-C: high-density lipoprotein cholesterol; WBC: White Blood Cell; PMN: polymorphonuclear neutrophil.

Significant values are in bold.

Characteristics of patients based on ACR and mortality status after liver transplantation are also indicated in Tables 3 and 4, respectively.

**Table 3:** Characteristics of cirrhotic patients based on acute cellular rejection (ACR) status after liver transplantation.

	Groups, mean ± SD		*P* value^a^
	ACR (no, n = 261)	ACR (yes, n = 73)	
Age, y	41.31 ± 18.97	38.89 ± 19.30	0.764
Male, n (%)	160 (61.3)	47 (64.3)	0.617
BMI ^b^, kg/m^2^	23.16 ± 4.71	23.64 ± 5.04	0.767
MELD-Na	20.31 ± 8.77	20.63 ± 10.08	0.269
Waiting time for transplantation, months	4.16 ± 5.40	4.70 ± 6.77	0.186
Cause of cirrhosis, n (%)			
Viral	35 (13.4)	11 (15.0)	0.472
NASH	39 (14.9)	9 (12.3)	
PSC/PBC	66 (25.2)	22 (30.1)	
AIH	38 (14.5)	8 (10.9)	
Genetic causes	27 (10.3)	8 (10.9)	
Other	56 (21.4)	15 (20.5)	
Alcohol intake (yes), n (%)	11 (4.2)	8 (10.9)	0.330
Smoking (yes), n (%)	1 (0.3)	1 (1.3)	0.816
CMV positive (yes), n (%)	28 (10.7)	36 (49.3)	**0.009**
Hypertension (yes), n (%)	16 (6.1)	8 (10.9)	0.396
Diabetes millitus (yes), n (%)	61 (23.3)	15 (20.54)	0.712
Medical condition at the time of LT, n (%)			
ICU	7 (2.6)	6 (8.2)	**0.022**
Hospitalized	73 (27.9)	21 (28.7)	
Home	181 (69.3)	46 (63.0)	
ALT (U/L)	101.87 ± 398.53	79.63 ± 106.94	0.361
AST (U/L)	121.79 ± 374.23	119.32 ± 187.26	0.959
ALP (U/L)	488.61 ± 368.32	508.06 ± 443.04	0.086
GGT (U/L)	142.46 ± 204.89	142.26 ± 142.63	0.126
Albumin (g/dL)	3.39 ± 0.78	3.34 ± 0.67	0.514
FBS (mg/dL)	111.40 ± 62.83	116.65 ± 60.52	0.478
TC (mg/dL)	144.54 ± 83.68	136.96 ± 95.03	0.867
TG (mg/dL)	119.77 ± 99.63	121.42 ± 81.68	0.749
LDL-C (mg/dL)	85.69 ± 57.58	81.56 ± 73.91	0.849
HDL-C (mg/dL)	34.56 ± 15.66	30.88 ± 13.43	0.332
WBC (per microliter)	6657.96 ± 3417.65	6779.71 ± 4374.85	0.050
PMN (%)	62.40 ± 14.74	64.81 ± 16.13	0.288
Lymphocytes (%)	25.86 ± 13.73	24.30 ± 13.81	0.938

^a^Obtained from Independent sample T-Test for continuous variables and Chi-square for Categorical variables.

BMI: body mass index; MELD-Na: Model For End-Stage Liver Disease; NASH: nonalcoholic steatohepatitis; PSC: primary sclerosing cholangitis; PBC: primary biliary cholangitis; AIH: autoimmune hepatitis; CMV: Cytomegalovirus; ICU: intensive care unit; ALT: alanine transaminase; AST: aspartate aminotransferase; ALP: alkaline phosphatase; GGT: gamma-glutamyl transferase; FBS: fasting blood sugar; TC: total cholesterol; TG: triglycerides; LDL-C: low-density lipoprotein cholesterol; HDL-C: high-density lipoprotein cholesterol; WBC: White Blood Cell; PMN: polymorphonuclear neutrophil.

Significant values are in bold.

**Table 4:** Characteristics of cirrhotic patients based on mortality status after liver transplantation.

	Groups, mean ± SD		*P* value^a^
	Mortality (no, n = 280)	Mortality (yes, n = 54)	
Age, y	38.88 ± 19.67	47.11 ± 14.58	**< 0.001**
Male, n (%)	173 (62.0)	34 (63.0)	0.895
BMI ^b^, kg/m^2^	23.06 ± 4.93	24.78 ± 4.08	0.398
MELD-Na	19.74 ± 8.61	23.82 ± 12.05	**0.045**
Waiting time for transplantation, months	4.12 ± 5.26	5.66 ± 9.02	**0.002**
Cause of cirrhosis, n (%)			
Viral	39 (14.0)	7 (13.0)	0.106
NASH	38 (13.6)	10 (18.5)	
PSC/PBC	75 (26.9)	12 (22.2)	
AIH	34 (12.2)	12 (22.2)	
Genetic Causes	34 (12.2)	1 (1.9)	
Other	59 (21.1)	12 (22.2)	
Alcohol intake (yes), n (%)	17 (6.1)	1 (1.9)	0.209
Smoking (yes), n (%)	1 (0.4)	0 (0.0)	0.660
CMV Positive (yes), n (%)	52 (18.7)	12 (22.2)	0.549
Hypertension (yes), n (%)	19 (6.9)	6 (11.5)	0.246
Diabetes millitus (yes), n (%)	63 (22.9)	13 (25.5)	0.689
Medical condition at the time of LT, n (%)			
ICU	4 (1.4)	6 (11.1)	**< 0.001**
Hospitalized	49 (17.5)	13 (24.1)	
Home	227 (81.1)	35 (64.8)	
ALT (U/L)	91.57 ± 334.16	98.13 ± 149.06	0.792
AST (U/L)	117.22 ± 315.63	139.30 ± 275.63	0.539
ALP (U/L)	491.92 ± 375.32	530.13 ± 513.42	0.080
GGT (U/L)	140.75 ± 182.54	180.43 ± 203.58	0.386
Albumin (g/dL)	3.42 ± 0.74	3.13 ± 0.71	**0.011**
FBS (mg/dL)	109.77 ± 57.20	132.60 ± 78.87	**0.030**
TC (mg/dL)	147.16 ± 91.21	91.17 ± 31.50	0.084
TG (mg/dL)	121.23 ± 94.92	116.17 ± 71.59	0.456
LDL-C (mg/dL)	87.57 ± 66.73	49.82 ± 20.53	0.076
HDL-C (mg/dL)	33.61 ± 15.15	28.09 ± 11.37	0.337
WBC (per microliter)	6623.93 ± 3710.72	7059.44 ± 4475.06	**0.043**
PMN (%)	61.80 ± 15.25	71.37 ± 13.23	0.495
Lymphocytes (%)	26.70 ± 14.03	17.80 ± 9.27	**0.008**

^a^Obtained from Independent sample T-Test for continuous variables and Chi-square for Categorical variables.

BMI: body mass index; MELD-Na: Model For End-Stage Liver Disease; NASH: nonalcoholic steatohepatitis; PSC: primary sclerosing cholangitis; PBC: primary biliary cholangitis; AIH: autoimmune hepatitis; CMV: Cytomegalovirus; ICU: intensive care unit; ALT: alanine transaminase; AST: aspartate aminotransferase; ALP: alkaline phosphatase; GGT: gamma-glutamyl transferase; FBS: fasting blood sugar; TC: total cholesterol; TG: triglycerides; LDL-C: low-density lipoprotein cholesterol; HDL-C: high-density lipoprotein cholesterol; WBC: White Blood Cell; PMN: polymorphonuclear neutrophil.

Significant values are in bold.

A significant difference was observed between the percentage of patients with hypertension and medical conditions at the time of LT between two groups of patients with and without ACR after LT. Furthermore, the findings of Table 4 showed that age, MELD-Na score, waiting time for LT, FBS, and WBC levels were significantly higher in patients with post-transplant mortality than the other group. However, albumin levels and the percentage of lymphocytes were lower.

The ORs (95% CIs) for ACR and HRs (95% CIs) for mortality after LT based on the pre-transplantation serum vitamin D level are reported in Tables 5 and 6, respectively. In the crude and first adjusted model (based on age, sex, and BMI), there was a significant association between vitamin D deficiency in the pre-transplant time and odds of ACR (odds ratio [OR] = 2.71, 95% confidence interval [CI] 1.61–4.29, *P* for trend < 0.001; OR = 2.68, 95% CI 1.63–4.39, *P* for trend < 0.001, respectively). Furthermore, in model 2, after adjusting for further confounders (waiting time for transplantation, medical condition at the time of LT, causes of cirrhosis, smoke, alcohol, hypertension, diabetes mellitus, and infection of CMV) and final model (further adjustment for MELD-Na, AST, ALT, PMN, WBC, and lymphocytes), vitamin D deficiency was associated with 2.68- (OR: 2.68; 95% CI 1.59–4.71, *P* for trend = 0.001) and 2.69-fold (OR: 2.69; 95% CI 1.50–4.68, P for trend = 0.001) increase in the odds of ACR, respectively.

**Table 5:** Odds ratio (OR) and 95% confidence interval (CI) for acute cellular rejection (ACR) of liver transplantation (LT) based on the serum vitamin D level of pre-transplantation.

	Vitamin D sufficiency (≥ 20 ng/ml)	Vitamin D deficiency (< 20 ng/ml)	Ptrend
ACR			
Crude model	1.00 (Ref)	2.71 (1.61–4.29)	**< 0.001**
Model 1*	1.00 (Ref)	2.68 (1.63–4.39)	** < 0.001**
Model 2^†^	1.00 (Ref)	2.68 (1.59–4.71)	**0.001**
Model 3^‡^	1.00 (Ref)	2.69 (1.50–4.68)	**0.001**

**Binary logistic regression was used to obtain OR and 95% CI.

*Model 1: adjusted for age, sex, and BMI.

^†^Model 2: Model 1 + adjusted for waiting time for transplantation, medical condition at the time of LT, causes of cirrhosis, smoke, alcohol, hypertension, diabetes mellitus, and infection of CMV.

^‡^Model 3: Model 2 + MELD-Na, AST, ALT, PMN, WBC, and lymphocytes.

Significant values are in bold.

**Table 6:** Hazard ratio (HR) and 95% confidence interval (CI) for mortality after liver transplantation (LT) based on the serum vitamin D level of pre-transplantation.

	Vitamin D sufficiency (≥ 20 ng/ml)	Vitamin D deficiency (< 20 ng/ml)	Ptrend
Mortality			
Crude model	1.00 (Ref)	2.64 (1.42–4.33)	**0.022**
Model 1*	1.00 (Ref)	2.02 (1.04–3.93)	**0.038**
Model 2^†^	1.00 (Ref)	1.63 (0.80–3.31)	0.177
Model 3^‡^	1.00 (Ref)	1.46 (0.71–3.00)	0.309

**Cox regression was used to obtain HR and 95% CI.

*Model 1: adjusted for age, sex, and BMI.

^†^Model 2: Model 1 + adjusted for waiting time for transplantation, medical condition at the time of LT, causes of cirrhosis, smoke, alcohol, hypertension, diabetes mellitus, and infection of CMV.

^‡^Model 3: Model 2 + MELD-Na, AST, ALT, PMN, WBC, and lymphocytes.

Significant values are in bold.

Although in the crude and first adjusted model, vitamin D deficiency in the pre-transplant stage was significantly associated with an increase in the risk of mortality after two years follow-up in cirrhotic patients undergoing transplantation (HR = 2.64, 95% CI 1.42–4.33; *P* for trend = 0.022; HR = 2.02, 95% CI 1.04–3.93, *P* for trend = 0.038, respectively), in the second and final adjusted model, no significant relationship was observed (HR = 1.63, 95% CI 0.80–3.31, *P* for trend = 0.177; HR = 1.46, 95% CI 0.71–3.00; *P* for trend = 0.309, respectively).

**Figure 1:**
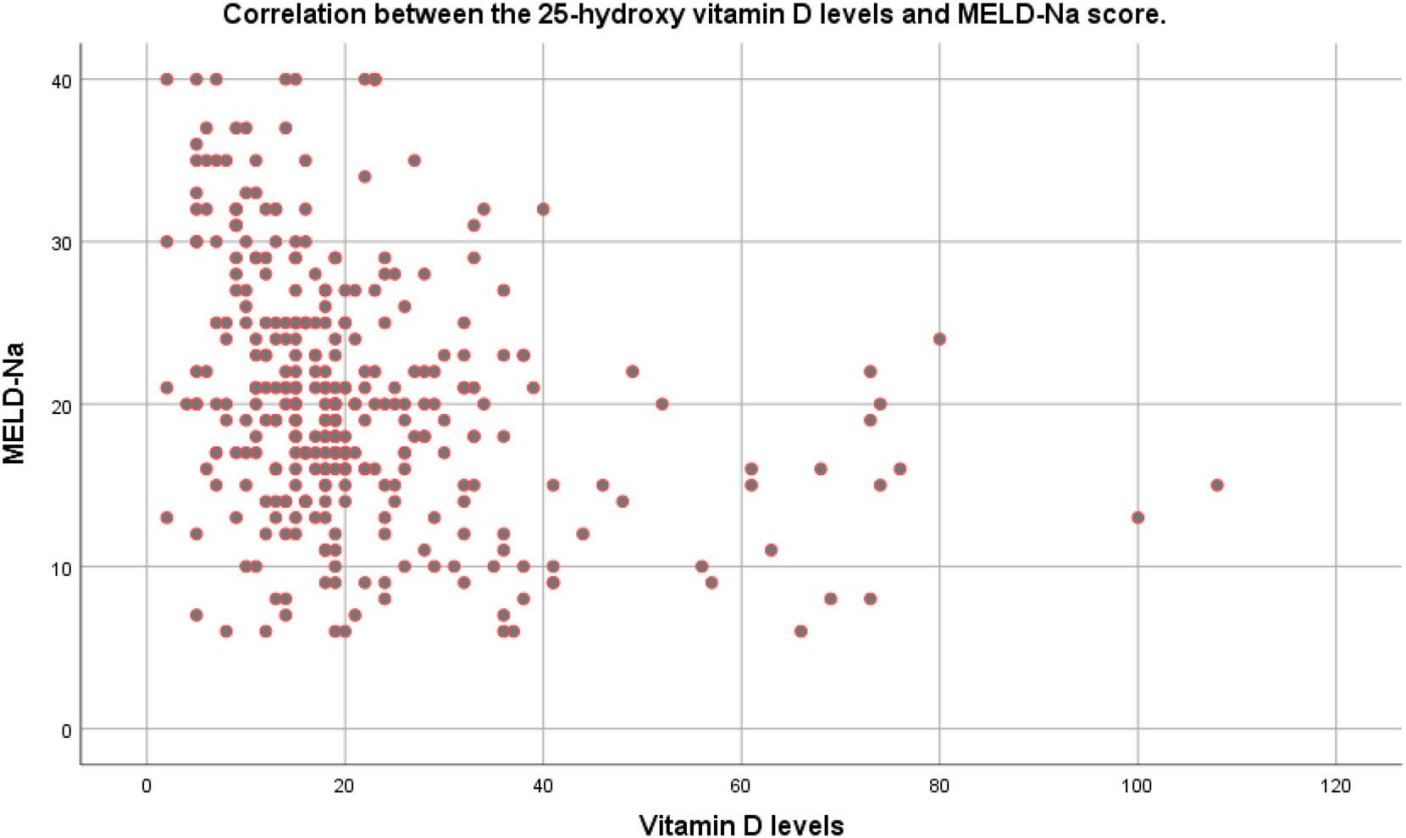
Correlation between the 25-hydroxy vitamin D levels and MELD-Na score. The correlation coefficient r was − 0.157 (*P* < 0.001; 95% CI: − 0.210, − 0.104).

The relationship between the MELD-Na score and serum vitamin D levels in the pre-transplantation period is demonstrated in Fig. 1. The findings suggested an inverse relationship between these two. The correlation coefficient r was − 0.157 (*P* < 0.001; 95% CI  − 0.210, − 0.104).

## Discussion

The current retrospective cohort study investigated the association between vitamin D deficiency prior to LT with ACR and mortality in liver transplant recipients. The study's findings indicated a significant correlation between vitamin D deficiency and increased odds of ACR after LT. Our finding is consistent with the investigation conducted by Doi et al., which demonstrated an association between vitamin D deficiency and a greater incidence of ACR in the short-term following LT compared to individuals with adequate vitamin D levels^11^. Zhou et al. also reported that a high 25(OH)D level (> 25 ng/mL) in the pre-transplant period is significantly associated with a lower ACR within 30 days following LT^17^. Furthermore, another study by Bitetto et al. corroborated these findings, establishing an independent association between low serum 25-hydroxyvitamin D levels (< 5 ng/mL) and the occurrence of moderate to severe ACR episodes within two months following LT^26^. Generally, these findings suggest that maintaining adequate levels of vitamin D during the pre-transplantation period plays a pivotal role in minimizing the risk of ACR following transplantation. Aside from this, some research has shown that vitamin D supplementation is associated with a lower risk of ACR^11,17,27^. According to a study by Grant et al., supplementation with cholecalciferol (vitamin D3) for 12 weeks after LT increased vitamin D levels and reduced ACR and infection rates^27^. Hence, vitamin D deficiency can potentially affect ACR, and its correlation cannot only explained by deterioration in liver function.

Additionally, we found that patients with vitamin D deficiency had higher scores on the MELD-Na than those with sufficient levels of vitamin D. This negative correlation has also been reported by Doi et al.^11^. MELD-Na/MELD scores are widely used to assess liver disease severity and predict mortality in individuals with cirrhosis^28^. Thus, vitamin D deficiency may be associated with the severity of cirrhosis and adversely affect LT outcomes. Nevertheless, it should be noted that malnutrition is common among patients with ESLD^29^, and it is well documented that malnourished ESLD patients exhibit a high prevalence of vitamin D deficiency^20,21,30^. Malnutrition per se can have a negative effect on survival^31,32^. Hence, serum 25-hydroxyvitamin D levels may function as a prognostic marker for morbidity and mortality.

Our retrospective cohort study also demonstrated a significant correlation between pre-transplantation vitamin D deficiency and an elevated mortality risk after transplantation in both the crude and first-adjusted models. Nevertheless, this association was not statistically significant in the second and final adjusted models, suggesting that other potential confounding variables may impact the relationship between vitamin D deficiency and mortality in cirrhotic patients undergoing transplantation. Similarly, Doi et al. reported no significant disparity in overall survival between individuals with vitamin D deficiency and those with sufficient vitamin D levels before LT. However, a notable discrepancy was observed in post-transplantation periods, suggesting that vitamin D supplementation or maintaining adequate vitamin D levels following transplantation may be of benefit^11^. In addition, it has been shown that vitamin D supplementation during the first month after LT is associated with a better survival rate than the control group^17^.

Several mechanisms may explain the protective effect of vitamin D, including decreased production of inflammatory mediators such as interleukin (IL)-2, IL-17, and interferon-gamma (IFN-γ)^33–35^, decreased dendritic cells (DCs) maturation^36^, and an increase in Treg cell immunoprotective activity^37^. Treg are immune-suppressive cells that suppress the immune system, maintain self-tolerance, and prevent autoimmune diseases^38^. Vitamin D has been shown to manipulate monocytes and dendritic cells at different levels, allowing these cells to exert tolerogenic effects^39^. Additionally, vitamin D can inhibit macrophage transition to the M1 phenotype and promote macrophages with M2 phenotypes^40^. M1 macrophages secrete inflammatory cytokines that hinder cell proliferation, potentially resulting in tissue damage, while M2 macrophages facilitate cell proliferation and tissue regeneration^41^. Furthermore, vitamin D is thought to inhibit the Nuclear factor kappa-light-chain-enhancer of activated B cells (NF-kB) pathway and cyclooxygenase (COX)-2 transcription^42^, leading to a reduction in reactive oxidative species (ROS) that can cause oxidative stress^43^, DNA damage, and cellular death.

Moreover, research has suggested that bacterial infections may serve as a possible trigger for transplant organ rejection^44^. Vitamin D is known to preserve the integrity of the intestinal barrier by upholding the expression of immune cells within the tight junction, which is crucial for impeding bacterial invasions^45^. Besides, vitamin D receptors are also present within the gut barrier, promoting the production of antimicrobial peptides (AMPs)^46^ and Claudin-5 expression^45^. The AMPs play a critical role in protecting against infection and innate immunity^47^. In addition to their broad-spectrum antimicrobial activity, these peptides possess diverse mechanisms of action and regulate the composition of the gut microbiome^48^. Claudin-5, a tight junction protein, plays a key role in maintaining the integrity of the bowel's physical barrier, and its disruption is directly related to intestinal inflammation^49^.

The present retrospective cohort study was conducted in two referral centers, which is a notable strength of the study. Additionally, the study had a relatively large sample size and included a comprehensive assessment of potential confounders while adjusting for multiple models, which further strengthens our findings. However, it is important to consider several limitations when interpreting the results. First, the study's retrospective design limits its ability to establish a causal relationship between vitamin D deficiency and ACR or mortality. Second, the study did not consider the effects of vitamin D supplementation, which may have affected the findings. Third, the characteristics of the donors were not included in the analysis, which could have impacted the results. Finally, we were not able to include immunosuppressant doses, which are used after transplantation, in our analysis.

## Conclusion

The present study provides evidence that pre-transplant serum vitamin D levels may predict ACR in patients with cirrhosis undergoing LT. The findings of this study may have important implications for clinical practice, and further research is needed to fully understand the potential benefits and risks of vitamin D supplementation in these patients.

## Data Availability

The datasets used and/or analysed during the current study available from the corresponding author on reasonable request.
